# Computed Cardiopulmonography for the Detection of Early Smoking-Related Changes in the Lungs of Young Individuals Who Smoke

**DOI:** 10.1016/j.chest.2024.02.006

**Published:** 2024-02-10

**Authors:** Jennifer L. Redmond, Fiona Kendall, Nicholas M.J. Smith, Snapper R.M. Magor-Elliott, Rob J. Hallifax, Christopher J. Fullerton, Graham Richmond, John H. Couper, Grant A.D. Ritchie, Peter A. Robbins, Nayia Petousi, Nick P. Talbot

**Affiliations:** a Department of Chemistry, Physical and Theoretical Chemistry Laboratory, University of Oxford, Oxford, England; b Department of Physiology, Anatomy and Genetics, University of Oxford, Oxford, England; c Nuffield Department of Clinical Medicine, University of Oxford, Oxford, England; d Oxford NIHR Biomedical Research Centre, University of Oxford, Oxford, England

To the Editor:

Smoking is the major risk factor for COPD, which affects > 300 million people and is a leading cause of death and disability.^1^ Currently, diagnosis relies on spirometry, which includes a reduced (< 0.70) ratio of FEV_1_ to FVC. These indexes are sensitive to large airway function, but the early adverse effects of smoking occur mainly in the small airways and include bronchoconstriction, inflammation, mucus hypersecretion, and loss of small airways.^2^ By the time a diagnosis of COPD can be made with spirometry, irreversible disease is usually present, and the opportunity for early intervention has often been missed. Accordingly, an international commission recently concluded that reliance on spirometry for COPD diagnosis was “flawed and dangerous” and called for more sensitive approaches.^1^

The onset and development of respiratory disease is unlikely to be evenly distributed within the lungs. Early airways disease is likely to cause changes in gas exchange inhomogeneity that should be detectable much earlier than changes in indexes of overall lung function, such as FEV_1_. On this basis, we have developed a new noninvasive method, called computed cardiopulmonography, for measuring lung inhomogeneity. This uses a novel highly accurate gas analyzer based on laser absorption spectroscopy, the molecular flow sensor, and a computational model of gas exchange.^3^^,^^4^ The model returns novel indexes of lung function that are highly abnormal in patients with established COPD and more sensitive than FEV_1_ in those individuals with early-stage disease.^4^^,^^5^ We hypothesized that this method would be sensitive enough to identify abnormal lung physiology in young healthy people who smoke.

## Methods

To test this hypothesis, 23 people who self-reported current cigarette smoking but have no history of cardiorespiratory disease (mean age, 25 ± 5 years [range, 19-32 years], 14 male individuals; BMI, 24 ± 4 kg/m^2^; smoking history mean ± SD, 4.3 ± 4.3 pack-years) and 23 age-matched and sex-matched healthy self-reported nonsmoking participants (age, 25 ± 5 years [range, 19-34 years]; 14 male individuals; BMI, 21 ± 3 kg/m^2^) performed standard spirometry and novel lung inhomogeneity testing. The study was approved by the South Central Oxford-A Research Ethics Committee (17/SC/0172). All participants gave written, informed consent.

The lung inhomogeneity test and related indexes have been described previously.^4^^,^^5^ Briefly, participants breathed through a mouthpiece with their nose occluded for 15 min. For 10 min, they breathed air; for the final 5 min, they breathed oxygen. Throughout, the molecular flow sensor measured the flux of respiratory gases at the mouth every 10 milliseconds. A mathematical model was fitted subsequently to the gas profile, which quantifies inhomogeneity by allowing 125 theoretic lung units each to receive a different fractional share of total lung compliance (CL), blood flow (Cd), and respiratory dead space (VD). A nonlinear least squares optimization algorithm was used to estimate the distribution of these parameters across the lung units, assuming a bivariate log-normal distribution for CL and Cd, and a normal distribution for VD. The SDs of each distribution (σlnCL [SD for the natural logarithm of standardized lung compliance], σlnCd [SD for the natural logarithm of the standardized vascular conductance], σVD [SD of the standardized dead space]) are the key model outputs and represent novel indexes of ventilatory, pulmonary vascular, and dead space inhomogeneity, respectively. The model also estimates functional residual capacity (FRC) and total VD.

To explore the effect of smoking on FEV_1_ percent predicted and model parameters, a linear regression model was used of the form:

X=Intercept+αln(Height)+βln(Age)+γln(BMI)+δ“isFemale”+εpack−years,

where X is the parameter of interest. For FRC and VD, natural logarithms of their values were used. For FEV_1_ percent predicted, height, age, and sex were excluded, because these are used in calculating the prediction, which was based on the equations from the Global Lung Initiative.

## Results

**Figure 1:**
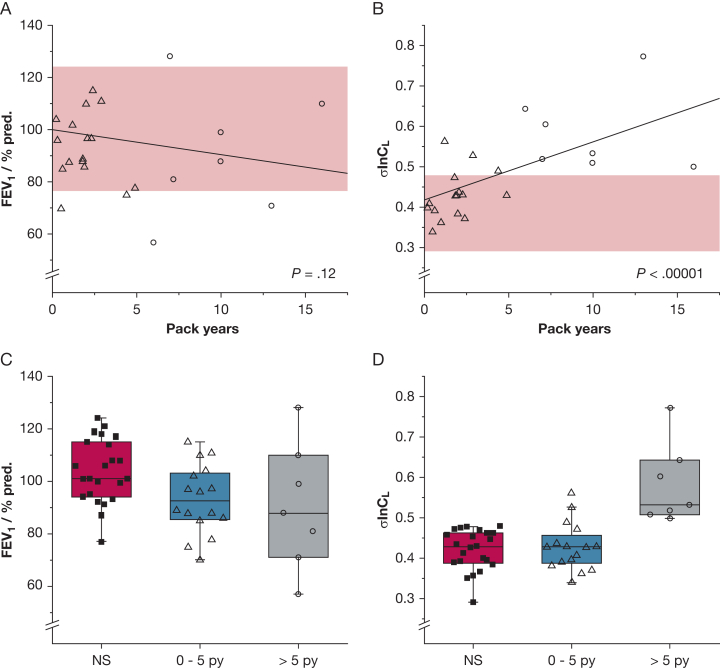
A-D, A, FEV_1_ percent predicted and B, σlnCL, respectively, for participants who smoke (n = 23) vs pack-y of smoking. Individual data points for nonsmoking participants are not shown, for clarity; however, the regression line and associated probability value were calculated with the use of data from both participants who smoke and nonsmoking participants (total n = 46). The shaded area indicates the range of values obtained for the nonsmoking participants. Categoric view of C, FEV_1_ percent predicted and D, σlnCL, respectively, for nonsmoking participants (n = 23), for participants who smoke with a 0-5 pack-y history of smoking (n = 16), and for participants who smoke with > 5 pack-y history of smoking (n = 7). Boxes indicate the interquartile range; the horizontal lines indicate the median value. Whiskers extend to the lowest or highest data point that is within 1.5 times the interquartile range of the lower or upper border of the box. Note that, for σlnCL, every value in participants who smoke with a > 5 pack-y history is above every value for the nonsmoking participants. In all panels, open triangles represent participants with a 0-5 pack-y smoking history; circles represent those with > 5 pack-y history. NS = nonsmoking participant; py = pack-year.

Figure 1, A and B, show the results for FEV_1_ percent predicted and σlnCL, respectively. For FEV_1_ percent predicted, no effect of smoking was detected. In contrast, σlnCL increased with pack-years in a highly-significant (*P* < .00001) manner. This remained significant (*P* < .0001), even when those participants with a > 10 pack-year smoking history were excluded from the analysis. Figure 1, C and D, show the distribution of values for FEV_1_ percent predicted and σlnCL, respectively, for nonsmoking participants and for two groups of people who smoke. For FEV_1_ percent predicted, the range of values for nonsmoking participants and people who smoke was similar, whereas for σlnCL, the range of values for the 23 nonsmoking participants was very narrow, with every value falling below those for the participants with a > 5 pack-year smoking history.

There was no significant effect of smoking history on any other model parameters, with the exception of σlnCd (*P* = .02). This may be secondary to the increased ventilation inhomogeneity (σlnCL). Across all participants, values for σlnCd and σVD were 0.82 ± 0.32 and 0.43 ± 0.08, respectively. The FRC was 3.25 ± 0.77 L, and VD was 0.15 ± 0.04 L. Male sex and height were associated with larger values for FRC and VD; BMI was associated with significantly smaller values for FRC. Participants’ physical characteristics had no significant effects on σlnCL, σlnCd, and σVD, other than an increase in σVD associated with female sex.

## Discussion

The parameter σlnCL represents a novel index of ventilatory inhomogeneity, which is likely to reflect underlying inhomogeneity of lung compliance. Assuming that differences in σlnCL are related to smoking per se, rather than an unknown confounding factor in those who smoke, our approach suggests that σlnCL may be a highly sensitive measure that can detect smoking-related lung pathophysiology in those individuals with a smoking history of just 5 pack-years. Previously, Verbanck et al^6^ performed multiple breath washout in 169 participants who smoked grouped by smoking history. Using the parameters S_cond_ and S_acin_, which represent inhomogeneity in conductive airways and acinar lung volume, respectively, they reported abnormalities in participants who smoke with > 10 pack-years but could not distinguish participants who smoke with < 10 pack-years from nonsmoking participants. Similarly, Jetmalani et al^7^ used multiple breath washout and impedance oscillometry to identify at least one abnormal parameter in > 75% of participants who smoke with an 8 to 40 pack-year history but normal spirometry. However, neither study observed such a clear separation between participants who smoke and nonsmoking participants. As a novel index, there is no gold standard against which to compare σlnCL directly, but future studies will compare the sensitivity of this parameter with these existing measures of inhomogeneity.

In contrast to these results in healthy people who smoke, in patients with COPD, we previously reported substantial increases in σVD and VD,^4^^,^^5^ raising the possibility that sequential changes in the model parameters may reflect the sequential nature of the pathophysiologic processes underlying COPD. In keeping with this, Hogg^2^ reported that loss of terminal bronchioles precedes other changes, such as emphysema. An important question for the current study is whether changes in σlnCL reflect irreversible small airway loss or reversible pathophysiologic changes, such as inflammation and/or bronchoconstriction. This could be assessed in future studies of smoking cessation.

A key research goal is to identify patients who are at risk of COPD early in the disease process.^1^ Although a number of risk factors are associated with declining lung function at a population level (including smoking, eosinophilic inflammation,^8^ and the presence of symptoms^9^), they are not sufficiently predictive to guide management in individual patients. Given the importance of small airways in obstructive lung disease,^2^ measures focused on the small airways may perform better. The results from the present study and other studies^4^^,^^5^^,^^10^ suggest that σlnCL may identify patients in whom the pathological changes leading to COPD are already underway, creating an important opportunity for early therapeutic intervention.

## Funding/Support

This work was supported by the National Institute for Health Research (NIHR) Oxford Biomedical Research Centre (BRC). J. L. R. and N. M. J. S. were supported by the Engineering and Physical Sciences Research Council (EPSRC). S. R. M. M. was supported by the British Heart Foundation (BHF).

## Financial/Nonfinancial Disclosures

The authors have reported to *CHEST* the following: Oxford University Innovation, a wholly owned subsidiary of the University of Oxford, holds/has filed patents relating to the background IP for the technology used in this work. P. A. R., G. A. D. R., and J. H. C. have an interest in one or more of these patents. J. L. R. has received support from the EPSRC, UKRI, and Eurofins Foundation. N. M. J. S. has received support from the EPSRC. S. R. M. M. has received support from the British Heart Foundation. C. J. F. has received support from the NIHR Oxford Biomedical Research Centre (BRC). G. A. D. R. has received research funding from the EPSRC. P. A. R has received research funding from the NIHR Oxford BRC. N. P. has received support from the NIHR Oxford BRC and speaker fees from AstraZeneca. None declared (F. K., R. J. H., G. R., and N. P. T.).
